# Early Protective Role of Inflammation in Cardiac Remodeling and Heart Failure: Focus on TNFα and Resident Macrophages

**DOI:** 10.3390/cells11071249

**Published:** 2022-04-06

**Authors:** Sophie Besse, Sophie Nadaud, Elise Balse, Catherine Pavoine

**Affiliations:** INSERM, Institute of Cardiometabolism and Nutrition (ICAN), Sorbonne Université, UMR_S1166, F-75013 Paris, France; sophie.besse@sorbonne-universite.fr (S.B.); sophie.nadaud@sorbonne-universite.fr (S.N.); elise.balse@sorbonne-universite.fr (E.B.)

**Keywords:** inflammation, TNFα, resident macrophages, monocyte-derived macrophages, adaptive cardiac remodeling, heart failure, aging

## Abstract

Cardiac hypertrophy, initiated by a variety of physiological or pathological stimuli (hemodynamic or hormonal stimulation or infarction), is a critical early adaptive compensatory response of the heart. The structural basis of the progression from compensated hypertrophy to pathological hypertrophy and heart failure is still largely unknown. In most cases, early activation of an inflammatory program reflects a reparative or protective response to other primary injurious processes. Later on, regardless of the underlying etiology, heart failure is always associated with both local and systemic activation of inflammatory signaling cascades. Cardiac macrophages are nodal regulators of inflammation. Resident macrophages mostly attenuate cardiac injury by secreting cytoprotective factors (cytokines, chemokines, and growth factors), scavenging damaged cells or mitochondrial debris, and regulating cardiac conduction, angiogenesis, lymphangiogenesis, and fibrosis. In contrast, excessive recruitment of monocyte-derived inflammatory macrophages largely contributes to the transition to heart failure. The current review examines the ambivalent role of inflammation (mainly TNFα-related) and cardiac macrophages (Mφ) in pathophysiologies from non-infarction origin, focusing on the protective signaling processes. Our objective is to illustrate how harnessing this knowledge could pave the way for innovative therapeutics in patients with heart failure.

## 1. Introduction to Short-Term Adaptive Cardiac Remodeling and Transition to Heart Failure

Clinical studies have clearly established that any abnormal change in left ventricular (LV) geometry (concentric (thickening) or eccentric (dilation)) is associated with an increased risk of cardiovascular disease [1]. Cardiac hypertrophy, initiated by a variety of physiological or pathological stimuli (hemodynamic or hormonal stimulation or infarction), is a critical early adaptive compensatory response of the heart [2]. Development of either physiological or pathological hypertrophy depends both on the nature of upstream stimuli and associated signaling mechanisms as well as the duration of cardiac stress [3]. Concentric hypertrophy (elevated h/r geometric parameter: diastolic wall thickness to radius ratio, associated with an increase in heart weight) has been described as an early adaptive response to maintain a normal systolic function [4], as recently illustrated in Flamant et al. [5]. The ability of the myocardium to successfully compensate for and adapt to environmental stress ultimately determines whether the heart will decompensate and fail or conversely maintain preserved function [2]. Age, gender, increased blood pressure and body mass index are key clinical risk factors of dynamic worsening. Data in humans regarding the development of LV geometric pattern over time are relatively scarce. However, there is substantial evidence for a potential temporal sequence of transient concentric hypertrophy evolving over the long term toward eccentric hypertrophy, dilation, and the development of heart failure (HF) [6,7], as already suggested in animal studies [5].

Physiological hypertrophy (during development, pregnancy, or endurance training) is totally reversible, characterized by mild heart growth (10–20% higher than that of a normal heart), absence of fetal gene program reactivation, an increase in individual cardiomyocyte growth in both length and width, angiogenesis, and the absence of apoptosis and interstitial fibrosis [8]. In contrast, chronic cardiac hypertrophy will eventually progress into HF, arrhythmia, and sudden death, following associated induction of apoptotic and fibrotic responses and the disruption of coordinated tissue growth with angiogenesis [9,10,11,12]. Adaptative concentric LV hypertrophy is also observed during aging, even in apparently healthy individuals [13]. Moreover, the left atrium enlarges and increases in volume roughly 50% between the third and eighth decade [14], predisposing elderly subjects to atrial fibrillation (AF).

Of note, physical exercise has a protective effect on the heart, and endurance training improves cardiac performance in hypertensive rats by converting pathologic hypertrophy into a more physiologic hypertrophy associated with lower apoptosis and fibrosis and higher angiogenesis [15,16].

In case of HF, the heart is unable to pump the blood efficiently due to ineffective muscle contraction (systolic HF) or relaxation or filling abnormalities (diastolic HF). Based on the ejection fraction, HF patients can be classified into two major groups. Patients with impaired systolic function are categorized in HF with reduced ejection fraction (HFrEF), whereas patients with diastolic dysfunction, often characterized by normal systolic function, are classified in HF with preserved EF (HFpEF). HFpEF has emerged as a critical health problem. Its prevalence increases with aging, obesity, diabetes, and hypertension. For example, in the setting of normal aging, HFpEF is promoted by an impairment of ventricular compliance due to the expansion of myocardial fibrosis, and disturbances of calcium homeostasis in hypertrophied cardiomyocytes, leading to a delayed relaxation [17]. In these elderly patients, the contribution of atrial contraction to ventricular filling is enhanced but atrial contractility is impaired, increasing the risk of the development of HF [18].

In most cases, early activation of an inflammatory program reflects a reparative or protective response to other primary injurious processes [19]. Later on, regardless of underlying etiology, HF is always associated with both local and systemic activation of inflammatory signaling cascades [19,20,21]. The structural basis of the progression from well-compensated hypertrophy to pathological hypertrophy and HF is still largely unknown [22]. Therefore, a better understanding of cellular mechanisms elicited during early remodeling is necessary to prevent the progression to HF or favor recovery [23,24]. To achieve this goal, there is accumulating evidence that advances in understanding the role of inflammation in tissue remodeling are essential [25].The current review examines the ambivalent role of inflammation (mainly TNFα-related) and cardiac macrophages (Mφ) in pathophysiologies related to hypertension, aortic stenosis, genetic cardiomyopathies, and sepsis or aging, with a special focus on related protective signaling processes.

## 2. Cardiac Remodeling, HF, and Inflammation

The link between HF and inflammation was first recognized in 1990 by Levine [26]. Since then, proinflammatory cytokines have emerged as determinant factors for initiating, integrating, and maintaining the myocardial response to stress [2], and there is evidence of an ongoing inflammatory response in all the manifestations of clinical HF [27]. For example, activation of the NLRP3 inflammasome is important for severe pressure overload-induced myocardial remodeling [28]. Inhibition of NLRP3 signaling reverses transverse aortic constriction (TAC)-induced pathological remodeling by attenuating hypertrophy, inflammation, and fibrosis via inhibition of calcineurin and MAPK activities, thereby improving contractile function [29]. Similarly, with age, high levels of oxidative stress and associated tissue damage (including cell death and fibrosis) trigger an inflammatory response, which importantly contributes to atrial and ventricular remodeling [30,31,32,33].

The prevailing concept has long been that inflammation, like one of its master regulator tumor necrosis factor alpha (TNFα), is harmful and precipitates transition from early cardiac remodeling to HF. In support for this concept, dysregulated cytokines expression (sustained and excessive), e.g., cardiac targeted TNFα overexpression, is sufficient to produce injury and provoke overt cardiac decompensation [34,35,36,37,38,39]. Elevated levels of TNFα have been associated with HF [26,27,40,41,42] and a progressive increase in serum TNFα correlated with disease progression (according to the New York Heart Association classification) [43,44]. By using TNFα-knockout mice, Sun et al. have demonstrated that, in the pressure-overload TAC model, TNFα contributes to adverse cardiac remodeling [45]. Accordingly, a series of multicenter clinical trials have been conducted in HF patients using compounds that trap TNFα, comprising infliximab, an antibody directed to TNFα, and etanercept, a soluble recombinant receptor of TNFα. Surprisingly, outcomes were disappointing, leading at best to no benefit, and at worst to HF worsening [46,47,48,49]. This highlighted the revisited cytokine hypothesis of a long-term deleterious but potentially beneficial short-term impact of inflammation [43,49,50]. In fact, a growing body of evidence supports the notion that short-term low-level expression of pro-inflammatory molecules is beneficial and acts as an early warning system (review in Mann [2] and Sacks [51]). This literature reporting beneficial effects of inflammation in the early stages of cardiac injury offers novel insights [52,53]. Interestingly, clinical studies have suggested the potential adaptive role of TNFα in early cardiac remodeling showing that myocardial TNFα gene expression is significantly higher in patients with compensated aortic stenosis than in patients with decompensated stenosis [54] and that elevation of circulating TNFα is associated with concentric left ventricular remodeling [55].

**Figure 1:**
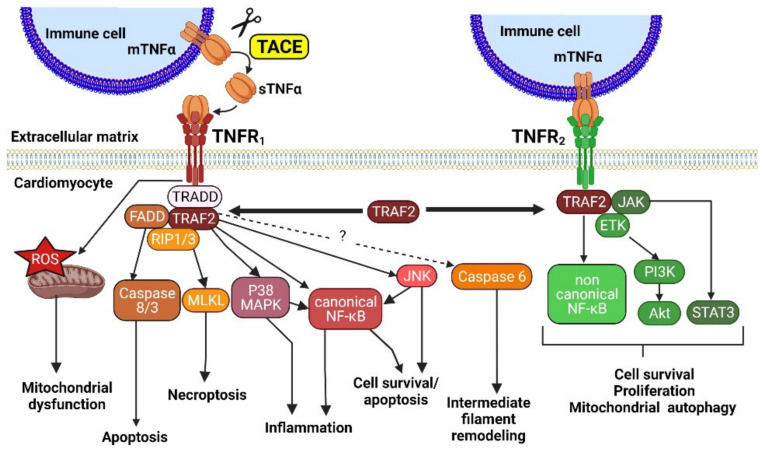
TNFα downstream signaling pathways mediated by the two receptors, TNFR_1_ and TNFR_2_. sTNFα, soluble tumor necrosis factor α; mTNFα, membranous tumor necrosis factor α; TACE, tumor necrosis factor α converting enzyme; TNFR_1_, Tumor necrosis factor receptor 1; TNFR_2_, tumor necrosis factor receptor 2; TRADD, TNFR_1_-associated death domain; FADD, fas-associated protein with death domain; RIP1/3, receptor interacting protein 1/3; ROS, reactive oxygen species; MLKL, mixed lineage kinase domain like pseudokinase; MAPK, mitogen-activated protein kinase; JNK, c-Jun N-terminal kinases; TRAF2, TNFR-associated factor 2; JAK, Janus kinase; ETK, epithelial and endothelial tyrosine kinase; PI3K, phosphoinositide 3 kinase; Akt, protein kinase B; STAT3, signal transducer and activator of transcription 3.

Figure 1 illustrates some of TNFα signaling pathways.

Since its discovery in 1975, the pro-inflammatory cytokine TNFα has been a subject of intense study [56]. TNFα is primarily produced by immune system cells, but also by all cell types in the heart, including cardiomyocytes. TNFα signals through two distinct membrane receptors, TNFR_1_ and TNFR_2_ [57]. TNFα exists as a membrane-bound form protein (mTNFα) that can be cleaved by a TNFα-converting enzyme (TACE) and released from cells as a soluble form of TNFα, sTNFα (Figure 1). The biological effects of sTNFα and mTNFα are not identical, with sTNFα and mTNFα preferentially activating TNFR_1_ and TNFR_2_, respectively [58]. Both receptors require the recruitment of adaptor molecules to initiate signaling, such as TNFR_1_-associated death domain (TRADD) and ubiquitin ligases and TNFR associated factors (TRAFs). TNFR_1_ and TNFR_2_ not only function independently, but also can influence each other via cross-talk between the different signaling pathways. A key player in TNFR_1_- and TNFR_2_-induced signaling is the RING finger protein TRAF2, which is recruited to both receptors upon their stimulation [59]. TRAF2 mediates cross-talk between TNFR_1_ and TNFR_2_, dictating the outcome of TNFα stimulation [59,60] (Figure 1). Membranous TNFR_1_ and TNFR_2_ can also be shed via cleavage by TACE and soluble truncated forms of TNFR_1_ and TNFR_2_ may lower the concentration of TNFα available for binding to functional cells [61,62].

### 2.1. TNFα, Early Adaptive Remodeling and HF

**Figure 2:**
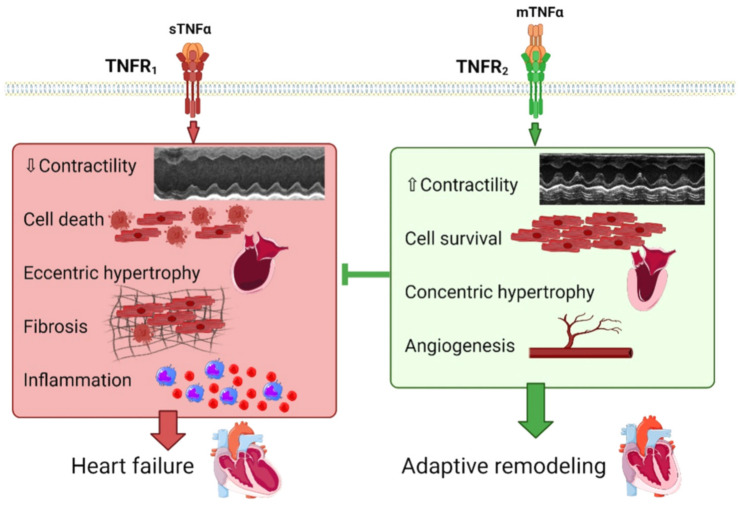
Physiopathological impact of TNFα signaling mediated by TNFR_1_ and TNFR_2_ on cardiac remodeling. sTNFα, soluble tumor necrosis factor α; mTNFα, membranous tumor necrosis factor α; TACE, tumor necrosis factor α converting enzyme; TNFR_1_, tumor necrosis factor receptor 1; TNFR_2_, tumor necrosis factor receptor 2. Macrophages are represented in blue and inflammatory monocytes in red (inflammation panel).

TNFα treatment has been shown to induce hypertrophy in isolated adult and neonatal cardiomyocytes, via ROS production, NFκB, MAPK and/or Akt signaling [63,64,65,66,67] (Figure 1). In line with this, TNFα overexpressing mice undergo ventricular hypertrophy, altered cardiac contractility and develop dilated cardiomyopathy [34,36,68,69], as illustrated in Figure 2.

**Figure 3:**
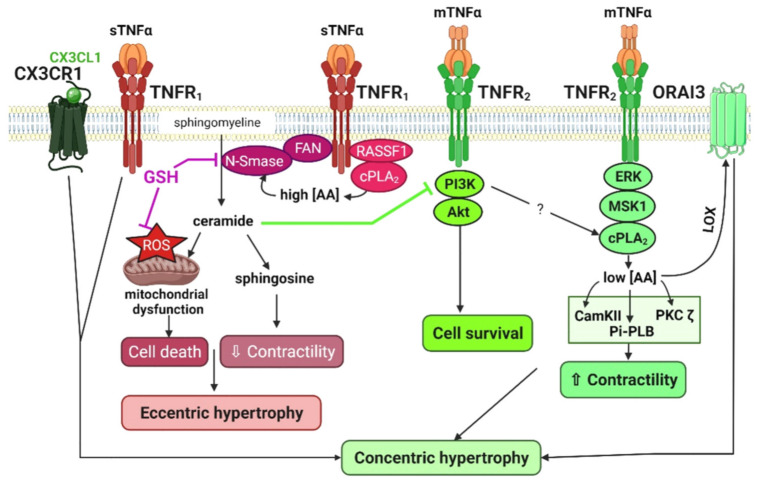
Determinant role of lipid signaling in TNFα–induced regulation of contractility, cell survival and hypertrophy. Synergistic action of TNFα and CX3CL1 drives a concentric hypertrophic response. sTNFα, soluble tumor necrosis factor α; mTNFα, membranous tumor necrosis factor α; TACE, tumor necrosis factor α converting enzyme; TNFR_1_, tumor necrosis factor receptor 1; TNFR_2_, tumor necrosis factor receptor 2; CX3CL1, fractalkine; GSH, glutathione; ROS, reactive oxygen species; FAN, factor associated with neutral sphingomyelinase activation; N-Smase, neutral sphingomyelinase; RASSF1, ras association domain family member 1; cPLA_2_, cytosolic phospholipase A2; AA, arachidonic acid; LOX, lipoxygenase, PI3K, phosphoinosisitde 3 kinase; Akt, protein kinase B; ERK, extracellular signal-regulated kinase; MSK1, mitogen- and stress-activated kinase 1; CamKII, calmoduline kinase II; PKC, protein kinase C; Pi-PLB, phosphorylated phospholamban.

In TNFα overexpressing mice, disruption of TNFR_1_ limits cardiac hypertrophic remodeling and preserves cardiac function [68]. The remaining hypertrophic response observed after TNFR_1_ ablation was suggested to be driven by TNFR_2_ [68]. By contrast, disruption of TNFR_2_ exacerbates dilation and HF [68]. In accordance, whereas cardiac restricted overexpression of mTNFα favors concentric hypertrophy that does not evolve towards dilated cardiomyopathy after 24 weeks, cleavable TNFα overexpression elicits a dilated cardiac phenotype [70,71] (Figure 2). Thus, interaction between mTNFα and TNFR_2_ may contribute to the beneficial effect of TNFR_1_ KO. In contrast, the interaction between sTNFα and TNFR_1_ may relay the deleterious effects of TNFR_2_ KO (Figure 2). In mice overexpressing cleavable TNFα, TACE inhibition abrogates the LV dilation and results in an increase in LV wall thickness, mimicking the effects observed in mice with non-cleavable mTNFα [68]. This suggests that posttranslational processing of TNFα is responsible for the dilated cardiac phenotype in mice with targeted cardiac overexpression of TNFα [70,71]. In addition, by using global KO mice or AAV9-mediated troponin C targeted deletion in cardiomyocytes, a recent study by Miao et al. demonstrates that transmembrane TNFα (mTNFα) attenuates pressure-overload TAC cardiac hypertrophy via TNFR_2_ [72] and suggests that preventing mTNFα cleavage by targeting the TNFα converting enzyme (TACE) rather than inhibiting TNFα signaling might be a valuable approach in HF [73]. TNFα signaling contributes to in vivo β-AR-mediated cardiac remodeling in a receptor-specific manner [74]. Unopposed TNFR_1_ activation is pro-inflammatory, pro-hypertrophic, and promotes functional decline. However, co-activation of TNFR_2_ during β adrenergic stress is anti-inflammatory and counterbalances these deleterious effects [74]. As proposed by Higuchi et al. [68], the opposite effects of TNFR_1_ and TNFR_2_ on cardiac remodeling and HF progression could rely on their opposite regulation of Akt, a pro-survival kinase, potently inhibited by the TNFR_1_-induced second messenger ceramide [75], as illustrated in Figure 3.

TNFα also impacts intermediate filament remodeling (Figure 1). TNFα has been reported to play a central role in end-stage HF in humans and mice, due to desmin (Des) cleavage by activated caspase 6 [76]. Des cleavage triggers aggregates formation, leading to intercalated disk destabilization, mitochondrial defects, cell death, and HF [77]. In TNFα overexpressing mice also expressing a caspase cleavage-resistant Des mutant (D263E), cardiac myocyte apoptosis was attenuated, LV wall thinning was prevented, and cardiac function was improved. This reveals an important role for Des cleavage in the development of TNFα-induced dilated cardiomyopathy and HF [77]. However, surprisingly, crossing the following two genetic HF models, namely TNFα overexpressing and Des^−/−^ mice, results in a considerable rescue of the typical Des^−/−^ extensive myocardial degeneration: mice display early cardiac hypertrophy, but prevention of adverse dilated remodeling and alteration of fractional shortening [78]. TNFα overexpression exerts a cardioprotective function through NF-κB-mediated cardiomyocyte ectopic expression of keratin 8 (K8) and keratin 18 (K18), two simple epithelia-specific intermediate filament (IF) proteins at the IDs [78]. The global nature of K8 and K18 ectopic protective induction was confirmed in stressed or failing cardiomyocytes by using experimental models of HF such as TAC or infarction, or in cardiomyocytes from human failing hearts, and associated with TNFα upregulation [78]. The mechanism of protection by TNFα through formation of a potential de novo alternative IF cytoskeletal system allowing to compensate for Des deficiency, could be through maintenance of mitochondrial function and intercalated disks integrity [78].

### 2.2. TNFR and NF-κB Signaling, Cell Survival and HF

NF-κB plays an essential role in cardiac remodeling and HF, essentially driven by two main pathways: the canonical pathway (involving p65, p50 and/or cRel protein members) and the non-canonical pathway (involving p52 and/or RelB) [79]. Activation of NF-κB relies on the nuclear translocation of homodimer or heterodimer forms of its members [79]. Increased activity and/or expression of NF-κB may participate in both cardioprotection (e.g., anti-apoptotic) [80,81], or in the development of heart diseases, as detailed in [79]. For example, the transition of cardiac hypertrophy to HF may be accompanied by NF-κB-mediated suppression of the sarcoplasmic reticulum Ca^2+^ ATPase 2 (SERCA2) transcription in ventricular myocytes [82]. NF-κB activation also mediates aging in the heart [83,84,85]. Different translocation patterns of NF-κB protein members were observed in aged murine models, but inhibition of NF-κB was generally suggested as protective [83,84,85]. In patients with valvular disease, higher NF-κB activity, higher TNFα levels, and more fibrosis characterized those with atrial fibrillation as compared to patients with sinus rhythm [86]. However, in other clinical studies, a loss of function mutation of NF-κB was considered either to confer susceptibility to left ventricular dysfunction [87], or to facilitate the onset of HF or worsen its prognosis [88]. These opposing findings suggest contrasting regulatory effects of the different NFkB members with complex outcomes.

TNFR_1_ is a death receptor, as its structure includes a death domain [60]. TNFR_1_ activates the canonical NF-κB pathway and the JNK/p38 MAP kinase pathway leading to either 1) inflammatory cytokines production or survival or 2) apoptosis or necroptosis, depending on receptor interacting protein 1/3 (RIP1/3) ubiquitination [60,80,89] (Figure 1). TNFR_2_ (lacking the death domain) can activate both the canonical NF-κB pathway (but to a lower extent as compared to TNFR_1_) and the non-canonical NF-κB pathway, mostly resulting in cell survival and proliferation [60]. A pro-survival signaling pathway termed the SAFE pathway (for survivor activating factor enhancement), involving TNFR_2_/STAT3 signaling, has also been identified to protect against MI [90]. The scaffolding protein TRAF2 may facilitate cytoprotective signaling downstream of both TNFR, playing a prosurvival key role to transduce activation of kinases and transcription factors [91,92,93] and promoting mitochondrial autophagy [94] (Figure 1).

### 2.3. TNFα, Contractile Function and HF

#### 2.3.1. The Neutral Sphingomyelinase, a Determinant of TNFR_1_ Deleterious Signaling

Lipid signaling plays a determinant role in TNFα-induced regulation of cardiac remodeling, as illustrated in Figure 3.

TNFα is essentially considered as a cardio-depressant mediator [95,96,97]. It has been shown to induce oxidant stress [98], to cause a drop in glutathione (GSH) levels, and to increase ceramide production through neutral-sphingomyelinase (N-Smase) activation (enzyme that converts sphingomyelin to ceramide). These mechanisms precede and regulate its depressant effects [95,99,100].

In control cardiac myocytes, TNFR_1_-dependent responses are predominant, overwhelming TNFR_2_ signaling but seem to be under the yoke of TNFR_2_, acting as a limiting factor [101]. TNFα exerts a dual positive and negative action on cell fractional shortening and alters cell survival [101,102,103,104,105]. The negative inotropic effect exerted by TNFα is thought to be mediated by TNFR_1_ [57,101,106]. In cardiomyocytes, activation of the N-Smase mediates TNFα-induced apoptosis and negative contractile effect [99,100,102,107]. This TNFα depressant effect is reproduced by sphingosine and suppressed by a specific inhibitor of ceramidase (enzyme that converts ceramide to sphingosine) [107] (Figure 3).

Glutathione is the physiological inhibitor of the neutral sphingomyelinase [99]. Administration to rats of the GSH precursor N-acetylcysteine (NAC) abrogates TNFα-induced N-Smase activation, oxidative stress, and negative effects on contraction in isolated cardiomyocytes [102]. One can speculate that glutathione status determines the adverse effects of TNFα in cardiac failure and that TNFα antagonism may be achieved by glutathione supplementation. In agreement, NAC, given orally as a curative treatment, replenishes cardiac GSH content, normalizes serum TNFα, and prevents morphological and functional cardiac injuries in the hypertensive high salt/L-NAME rat model. Of note, the NAC effect likely derives both from GSH-induced N-Smase direct inhibition [99,108] and from GSH anti-oxidant action [99] (Figure 3).

Treatment with a neutralizing anti-TNFR_1_ antibody or the GSH precursor, NAC, favors the emergence of the TNFR_2_ signaling, driving a positive effect on cell fractional shortening [101]. Thus, NAC treatment is proving a valuable anti-inflammatory tool to neutralize TNFR_1_-dependent signaling [101,102] and promote the emergence of TNFR_2_ pathways. In contrast, neutralizing anti-TNFR_2_ antibodies exacerbates TNFα-induced ROS production, negative inotropic impact and cell death, arguing for a protective role of the TNFR_2_ pathway and a TNFR_1_ and TNFR_2_ signaling interplay [101].

#### 2.3.2. The Cardiac cPLA_2_, a Determinant TNFR_2_ Protective Signaling Pathway: Involvement in β_2_-Adrenergic Signaling and Relationship with PI3Kinase Activity

Phospholipase A_2_ enzymes (PLA_2_s) catalyze the hydrolysis of the sn-2-fatty acyl ester bonds of membranous glycerophospholipids, leading to the liberation of lysophospholipids and free fatty acids including arachidonic acid (AA) [109,110].There is accumulating evidence for the determinant role of the cytosolic PLA_2_ (cPLA_2_)/AA pathway in cardiac TNFα signaling. AA activates N-Smase activity [111] (Figure 3). Thus, the TNFR_1_-induced negative contractile effect of TNFα is reproduced by high concentrations of AA [112]. In contrast, low concentrations of AA mediate TNFR_2_ signaling, leading to an improvement of the contractile function [108] (Figure 3). MacEwan’s group highlighted distinct regulations of the cPLA_2_ phosphorylation, proteolysis and activation by TNFR subtypes [113]. In addition, Mohamed et al. demonstrated the essential role of the RASSF1 (Tumor Suppressor Ras-Association Domain Family Protein 1A) adaptor protein in regulating downstream TNFα signaling via cPLA_2_ [103]. In adult rat cardiomyocytes, the study by Defer et al. identified a TNFR_2_-dependent activation of the cPLA_2_ together with the phosphorylation of ERK, MSK1, PKCζ, CAMKII, and phospholamban (Thr17 residue), leading to a positive action on calcium cycling and cell fractional shortening [101] (Figure 3).

Accumulating evidence highlights the cross-talk between inflammatory cytokines and sympathetic systems [61,114]. The sympathetic nervous system serves as one of the first mechanisms of compensation in response to cardiac injury. β-adrenergic receptor (β-AR) signaling defects are central features of human HF, with a selective decrease in β_1_-ARs number, and an impairment of the coupling of both β_1_-ARs and β_2_-ARs to Gs and adenylyl cyclase (AC) [115]. In a manner similar to TNFR_2_ signaling, the cPLA_2_ pathway has also been reported to play an important cardioprotective role in β_2_-AR signaling [116,117,118,119,120]. In homeostatic conditions, in embryonic chicks [117], and in adult rats [116] ventricular myocytes, β_2_-AR stimulation activates cPLA_2_/AA signaling, supporting calcium cycling and cell contraction. In the context of HF, the recruitment of the cPLA_2_ by β_2_-AR in the human heart has been evidenced in situations of altered β-AR (both β_1_-AR and β_2_-AR subtypes) coupling to AC/cAMP/PKA signaling [119]. Importantly, this suggested that cPLA_2_ signaling might compensate for impaired cAMP/PKA signaling occurring in aging or failing hearts [118,119]. In line with this, the group of Lipsius recently demonstrated that inhibition of PKA by phosphatidylinositol-3-kinase (PI3kinase) favors β_2_-AR stimulation of cPLA_2_ [121]. This study illustrates the potential association between cPLA_2_ signaling and activation of the PI3Kinase [121], a downstream target of β_2_-AR signaling initially identified by Xiao et al. [122] driving a strong cell survival signal in adult rat cardiomyocyte [123,124]. Similarly, the TNFR_2_ pathway has been associated not only with cPLA_2_ activation but also with PI3K stimulation [125]. Whether TNFR_2_-dependent activation of PI3Kinase favors cPLA_2_ signaling remains to be investigated.

By using cPLA_2_ knockout mice, the group of Bonventre and Force has shown that cPLA_2_ mitigates both normal and TAC-induced cardiac pathological hypertrophy, limiting growth factor IGF_1_ signaling, via AA-induced translocation to the membrane and activation of PKCζ and PDK1, pivotal players in cardiac hypertrophy [126,127]. However, cPLA_2_ metabolites have also been implicated as positive regulators of cardiac growth [128,129]. Concerning the TNFα signaling, the cPLA_2_ pathway also plays a role in modifications of Ca^2+^ handling remodeling that drive TNFα-protective hypertrophic and anti-apoptotic responses in hypertrophied cardiomyocytes [130,131] (Figure 3). Our group highlighted a TNFα/TNFR_2_-dependent signaling leading to ORAI3-dependent Ca^2+^ channel activation promoting early adaptive cardiac hypertrophy (ECH) and resistance to oxidative stress in rats subjected to isoproterenol infusion or abdominal aortic banding [130,131]. Of note, the regulation of ORAI3 by TNFα is detected in hypertrophied cardiomyocytes but not in normal counterparts. ORAI3-driven store-independent Ca^2+^ influx relies on cPLA_2_ activation [131], initial AA production and further AA metabolism via the lipoxygenase (LOX) pathway [131,132,133] (Figure 3). ORAI3 pharmacologic or molecular (siRNA) neutralization inhibits protective GSK3β phosphorylation, impairs early adaptive cardiac hypertrophy and accelerates HF [131].

### 2.4. Combined Signaling of TNFα with the CX3CL1 Chemokine

Unrelated to this previously identified TNFR_2_-ORAI3 pathway, our recent study shows that synergistic action of TNFα with the chemokine CX3CL1 promoted adaptive cardiac concentric hypertrophy in response to early β-AR chronic stimulation and limited transition toward eccentric dilated remodeling (low h/r geometric parameter) and HF [5]. This newly identified compensatory TNFα signaling relied on binding to TNFR_1_ (Figure 3). These results illustrated the protective role of the CX3CL1/CX3CR1 axis in early cardiac remodeling. Other studies have reported that CX3CL1 increases endothelial and smooth muscle cell migration and proliferation and acts as a proangiogenic factor that favors neovascularization [134]. Importantly, our results suggested the participation of TNFα, CX3CL1-cosecreting Mφ and their crosstalk with CX3CR1 expressing cardiomyocytes to delay HF [5].

## 3. Innate Immunity, Cardiac Remodeling and HF

Recent developments in the field of innate immunity have further advanced our understanding of the major role of inflammation in the pathogenesis of HF [27] or aging [135]. In particular, cardiac remodeling is a complex inflammatory syndrome where Mφ play a determinant role. Mφ reside in the tissue in the absence of injury and inflammation, but also play a major role following myocardial stress, where they can be protective or harmful [136,137]. Mφ influence tissue homeostasis, repair and regeneration in response to injury and modulating cardiac hypertrophy and HF [138,139,140]. These plastic cells adapt their physiology in response to cardiac and systemic stimuli. They are crucial in controlling and regulating the local tissue microenvironment, the matrix, oxygen content, acidification, and other molecular components (e.g., cytokines, growth factors, and chemokines) associated with micro-environmental shifts [141]. Mφ metabolism, including lipid metabolism, not only provides energy but also greatly influences Mφ phenotype and function, for example modulating signal transduction and gene regulation [142]. Dysregulation of lipid metabolism in Mφ is associated with various diseases [142].

Mφ have been extensively implicated in the inflammatory response to myocardial infarction (MI) [143]. A growing body of evidence suggests that they also play a critical role in the pathogenesis of chronic non-ischemic heart remodeling, e.g., after TAC [144,145,146,147].

Striking increases in the accumulation of recruited inflammatory Mφ in the heart within days to weeks following TAC, are linked to fibrosis and adverse LV remodeling [148,149]. In agreement, clodronate-induced Mφ depletion decreases infiltration of inflammatory Mφ and reduces LV hypertrophy in a model of hypertensive heart disease elicited by angiotensin II [150]. These studies are consistent with and further support the notion that inhibition of inflammatory signals is effective at preventing HF development after an increase in mechanical overload [28,147].

However, other studies have shown that the inflammatory response induced by the innate immune system can be physiological and results in the upregulation of cytoprotective responses that allow the heart to adapt to stress in the short term [2]. For instance, the study by Keck et al. points out inflammation arising from cardiac resident CD11b/c cells as a potential trigger of TNFR_2_- and ORAI3-dependent protective signaling pathways in cardiomyocytes, promoting early adaptive hypertrophy, improving resistance to oxidative stress, and delaying transition to HF, in response to TAC-induced pressure overload or β-adrenergic chronic infusion [131].

Therefore, cardiac Mφ are an emerging focus for therapeutic strategies aimed at strengthening adaptive responses, minimizing cardiomyocyte death, ameliorating pathological cardiac remodeling, and for treating HF [151].

## 4. Macrophages Subsets and Cardiac Remodeling

Metchnikov first described Mφ as phagocytic cells and key mediators in the phagocytosis theory in the late 1880′s [152] and received the Nobel prize in Physiology or Medicine for his work in 1908. Cardiac Mφ comprise 5–10% of total myocardial cells and are the most abundant leukocyte species in the heart [153,154]. In mice, their identification is based on “classical” surface markers (F4/80, CD64, CCR_2_, CX3CR1, MERTK, Ly6C, MHCII, CD206), novel markers (LYVE1 and TIMD4) or intracellular (CD68) molecule expression [155,156].

For years Mφ were thought to derive exclusively from circulating monocytes becoming tissue-resident after infiltration and differentiation [157]. We now know that many Mφ from embryonic origin integrate tissues prior to the onset of hematopoiesis [158,159]. Thus, cardiac tissue Mφ, either derive from embryonic origin independent of hematopoiesis (CCR_2_^−^/Ly6C^low^/MHCII^low/high^) and persist in adultwood through in situ proliferation, or originate from monocyte infiltration (CCR_2_^+^/Ly6C^high^/MHCII^high^) and replenish by circulating monocyte seeding [160]. Equivalent Mφ subpopulations (CCR_2_^−^ and CCR_2_^+^) were identified in the human heart [161]. During aging, the number of fetal liver-derived cardiac resident Mφ decreases and a substantial pool of adult cardiac Mφ is replenished by Mφ derived from bone marrow or spleen monocytes, suggesting an age-associated decrease in the local self-renewal capacity of resident CCR_2_^−^ Mφ [135,160,162].

Current knowledge gives clear evidence that the different cardiac Mφ populations are plastic, display various responses to injury, and differentially regulate repair processes. It thus appears that a timely planned targeting of specific subsets of Mφ will probably be necessary to achieve beneficial results in HF.

Strikingly, recent mapping and genetic depletion studies allowed to begin to decipher the functional roles of various Mφ populations and identify functions far beyond a phagocytic and immunologic role, e.g., maintaining mitochondrial function, facilitating cardiac conduction, and promoting coronary development and lymphangiogenesis [136,163].

**Figure 4:**
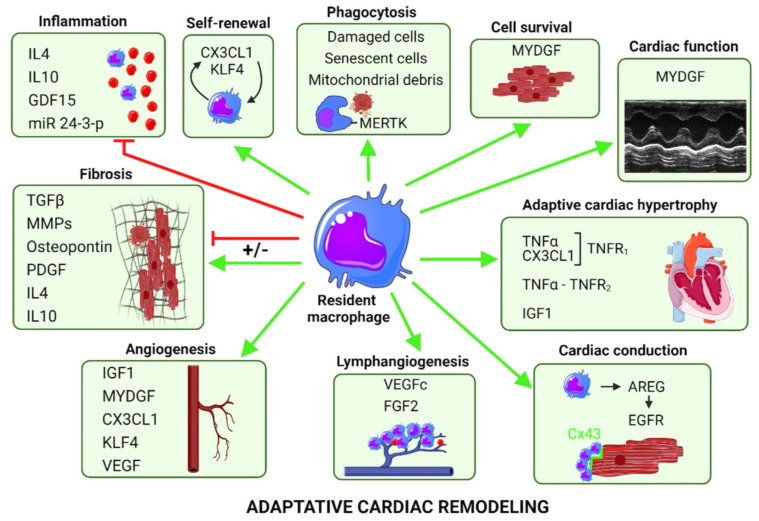
Induction of adaptive cardiac remodeling by resident CCR_2_^−^ Ly6C^low^ macrophages. CCR_2_, C-C motif chemokine receptor 2; Ly6C, lymphocyte antigen 6 complex; IL4, interleukin 4; IL10, interleukin 10; GDF15, growth differentiation factors 15; CX3CL1, fractalkine; KLF4, Kruppel-like factor 4; MERTK, myeloid-epithelial-reproductive tyrosine kinase; MYDGF, myeloid derived growth factor; IGF1, insulin-like growth factor-1; TGFβ, transforming growth factor β; MMPs, metalloproteinases; PDGF, platelet-derived growth factor; VEGF, vascular endothelial growth factor; FGF2, fibroblast growth factor 2; AREG, amphiregulin; EGFR, epidermal growth factor receptor; Cx43, connexin 43. Resident macrophages are represented in blue and inflammatory monocytes in red (inflammation and lymphangiogenesis panels). Positive (green line) and negative (red line) regulations.

Figure 4 illustrates the impact of cardiac resident Mφ in adaptive cardiac remodeling.

### 4.1. Resident Mφ Are Requisite for the Adaptive Response to Pressure Overload or Hypertension

An increase in pressure overload (e.g., TAC model) triggers an early concentric hypertrophic response of the myocardium. Cardiac-resident Mφ with low expression of Ly6C, generally considered as predominantly anti-inflammatory, were identified as critical mediators of this adaptive response by cardiomyocytes [164] (Figure 4). In contrast, a consensus seems to indicate that recruited pro-inflammatory CCR_2_^+^ Mφ, rather than resident CCR_2_^−^ Mφ, mediate pathological hypertrophy during the late phase of pressure overload [147,165,166].

**Figure 5:**
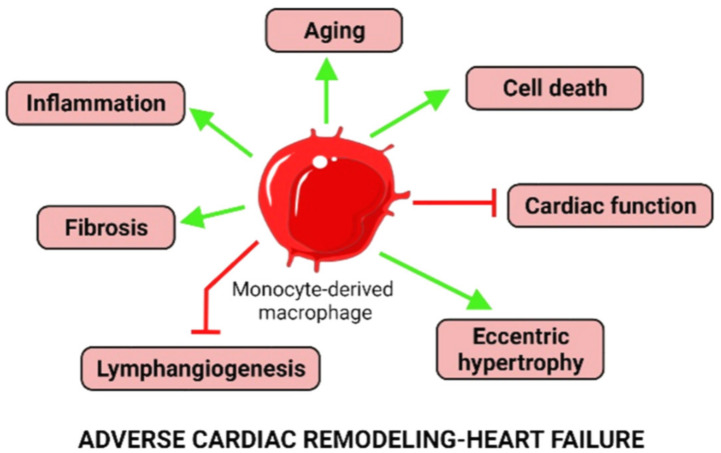
Induction of heart failure by monocyte-derived CCR_2_^+^ Ly6C^high^ macrophages. CCR_2_, C-C motif chemokine receptor 2; Ly6C, lymphocyte antigen 6 complex. Positive (green line) and negative (red line) regulations. Monocyte-derived inflammatory macrophage represented in red.

Global depletion of Mφ in the setting of hypertension worsens cardiac function but improves fibrosis suggesting dual protective and pathological functions of diverse Mφ populations [165,167,168,169]. Monocyte-derived Mφ (CCR_2_^+^) promote tissue damage and fibrosis in hypertension [170,171], as illustrated in Figure 5.

In contrast, a potential protective role of self-renewing resident-Mφ (CCR_2_^−^) has emerged from recent studies [137,156,169,172,173] (Figure 4).

#### 4.1.1. Protective Growth Factor Secretion by Resident Mφ

##### IGF1

Adaptive cardiomyocyte growth allows the myocardium to withstand hypertensive stress. Fate-mapping approaches, genetic ablation of resident Mφ or specific deletion of IGF1 in resident Mφ recently highlighted that the ability of the heart to adapt to hypertension is dependent on local IGF1 produced by resident Mφ [169]. Selective reduction of resident Mφ abolishes adaptive cardiomyocyte growth and leads to adverse remodeling (fibrosis), dilation, and severe cardiac dysfunction [128,129]. Of note, IGF1 was also previously identified as a potential mediator of the proangiogenic properties of embryonic-derived Mφ [174] (Figure 4).

In a mouse model of chronic dilated cardiomyopathy harboring a causative human mutation of the troponin T2 gene, Wong et al. demonstrate that CCR_2_^−^ Mφ, that interact with neighboring cardiomyocytes through focal adhesion complexes, sense mechanical stretch. This triggers their activation through a transient receptor potential vanilloid 4 (TRPV4)-dependent pathway and enhances their growth-factor expression, notably IGF1 [175]. This mechanism supports the determinant early protective role of CCR_2_^−^ Mφ in adaptive remodeling, coronary angiogenesis, cardiac output maintenance, and mice survival [175]. Wong et al. confirmed the CCR_2_^−^ Mφ-induced adaptive protection in a TAC model [175].

##### AREG/EGFR

The group of Manabe elegantly demonstrated that Ly6C^low^ Mφ, upregulated in the TAC model, secrete amphiregulin (AREG), which directly induces hypertrophy of neonatal cardiomyocytes in vitro [164]. In addition, AREG, produced by resident Mφ, controls connexin-43 (Cx43) phosphorylation and localization in cardiomyocytes, and therefore regulates cardiac impulse conduction [176] (Figure 4). The involvement of EGFR, a low-affinity receptor for AREG, and activation of a MEK/ERK pathway is suggested by Sugita et al. [176]. Thus, AREG is proposed as a potential therapeutical target for the prevention of arrhythmogenicity and sudden death after right ventricle or acute β adrenergic stress [176]. Notably, Son et al. describe a decrease in LV *Areg* mRNA expression in patients who suffered sudden cardiac death [177]. This points out a new protective mechanism in addition to the direct capacity of resident Mφ to connect to cardiomyocytes through Cx43-containing gap junctions that influences propagation of electrical signals and contributes to cardiac conduction in the AV node, previously identified by Hulsman et al. [178,179,180] (Figure 4).

However, activation of the AREG/AKT/mTOR pathway by using a chronic treatment with a GABA2R agonist has been shown to increase MHCII^high^ vs. MHCII^low^ Ly6C^low^ Mφ, and favor not only hypertrophy but also the development of cardiac fibrosis and the transition from concentric adaptive to eccentric remodeling. This suggested, in the long term, a potential participation of the pathway in cardiac decompensation and HF [181].

##### MYDGF

In contrast, some anti-hypertrophic inflammatory stimuli are beneficial and mediate adaptation to pressure overload [52]. By using the TAC model, the group of Wollert recently demonstrated that myeloid-derived growth factor (MYDGF), secreted by both CCR_2_^high^ and CCR_2_^low^ Mφ, attenuates LV hypertrophy and dysfunction via activation of the Pim1 proto-oncogene (PIM1) kinase and enhancement of SERCA2a expression [52,182]. Of note, MYDGF expression by both CCR_2_^high^ and CCR_2_^low^ subsets of Mφ is in line with the notion that these subsets exert distinct and partially overlapping functions [147,165,182,183]. MYDGF is a paracrine protein produced by bone marrow- and spleen- derived mononuclear monocytes and Ly6C^low^ cardiac Mφ, as initially demonstrated [184], but also by endothelial cells, as more recently suggested [185]. MYDGF reduces scar size and improves heart function after MI via the MAPK-STAT3 signaling pathways, favoring endothelial cell proliferation and angiogenesis and limiting cardiomyocytes apoptosis [184] (Figure 4). MYDGF also promotes post-MI heart regeneration in neonates and adults by favoring cardiomyocyte proliferation and expansion via the c-Myc/FoxM1 pathway [185]. MYDGF is a promising target to reverse cardiac remodeling and HF because, in mice models, recombinant MYDGF protein improves heart regeneration both in neonatal and adult heart after MI or TAC injury [182,184,185]. Of note, MYDGF levels are increased in both heart and plasma post-MI patients [184].

##### GDF15

GDF15 is a particularly interesting growth factor, described as protective in cardiovascular diseases. This cardiac-inducible factor is upregulated with aging or after various cardiovascular events linked to inflammation and oxidative stress [186] and is secreted by different cell types including cardiomyocytes and Mφ [187]. Exposure of Mφ to pro-inflammatory cytokines such as TNFα and TGFβ upregulates GDF15 expression [188]. Although GDF15 was reported to be induced in a pressure-overload murine model, its cardiac-specific overexpression antagonizes the hypertrophic response and the loss of ventricular performance [189]. GDF15 exerts anti-inflammatory effects by 1) limiting the recruitment of infiltrating pro-inflammatory cells through direct interference with chemokine signaling and integrin activation [190] and 2) promoting the M2 polarization of Mφ [191] (Figure 4). Chronic increase in circulating GDF15 levels have been reported both in HFpEF and HFrEF patients [187], and GDF15 has recently emerged as a strong and independent biomarker for identifying patients displaying HF with midrange or preserved EF with a worse prognosis [192].

##### VEGFc-d and FGF2

The lymphatic system has recently emerged as an important regulator of the interstitial fluid compartment, the immune cell transport and tissue remodeling during cardiac pathology and is under the control of Mφ populations [163,193,194]. A dysfunctional lymphatic system promotes exacerbation of chronic inflammation and long-term deterioration of cardiac function after MI [195]. Inversely, stimulation of lymphangiogenesis by VEGFc treatment after MI was found to reduce fibrosis and inflammation and to improve cardiac function [195]. A peculiar CCR_2_^low^ FLT_3_^low^ Mφ population (L^+^), that renews by in situ proliferation, and secretes pro-lymphangiogenic growth factors VEGFc-d and FGF2, has been identified in a TAC model and was shown to be associated with preservation of the lymphatic network during cardiac remodeling [163] (Figure 4). The lymphatic system’s ability to recruit and transport immune cells to drain lymph nodes during pressure overload depends on LYVE-1 expression on lymphatic endothelial cells, acting as a docking receptor for hyaluronic acid-coated leukocytes [163,195]. The reduction of CCR_2_-dependent monocyte recruitment during TAC using a CCR_2_ antagonist abrogates the loss of LYVE-1 on lymphatic endothelial cells, enhances L^+^ Mφ proliferation, reduces fibrosis, and improves cardiac function [163] (Figure 5).

In addition, VEGF secreted by resident Mφ is a well-known key mediator of angiogenesis, e.g., in response to TAC [172,173] (Figure 4).

We have recently performed a transcriptomic analysis comparing genes expressed by Mφ isolated from early compensated (ECH) or failing (HF) hearts [141]. Interestingly, we identified panels of hypertrophy-related genes selectively regulated in ECH Mφ (*Rcan1*, *Pik3ip1*) or HF Mφ (*Adam22*, *Tet2*, *Map3k2*, *Sik1*) and thus potentially associated with compensated or failing hypertrophy remodeling, respectively. In addition, ECH Mφ were characterized by an induction of *Egfr* mRNA expression, whereas HF Mφ displayed upregulated *Igfbp4* (insulin-like growth factor binding protein 4), a negative regulator of IGF1 signaling [141]. Such genomic or proteomic approaches may constitute the basis for future, more in-depth studies to identify important Mφ-related pathways interfering in cardiac remodeling as well as to characterize biomarkers associated with early vs. late disease progression [141,196,197,198,199].

### 4.2. Protective Phagocytic Activity of Cardiac Mφ

Overwhelming evidence from both preclinical and clinical studies indicates bioenergetics insufficiency in HF [200]. Thus, mitochondrial dysfunction seems to be an important target for therapy to directly improve cardiac function [200]. Interestingly, cardiac Mφ regulate myocardial homeostasis through effects on mitochondrial homeostasis [201]. MERTK expression is associated with anti-inflammatory and phagocytic Mφ functions. A recent study recently described that defective mitochondria debris are routinely ejected from cardiomyocytes as particules whose elimination is ensured by resident MERTK^+^ Mφ, enabling to preserve metabolic stability and ventricular function [201] (Figure 4).

With aging, cardiac cells that express senescence markers and display a so-called senescence-associated secretory phenotype (SASP) accumulate in the myocardium, [202,203]. To maintain tissue homeostasis, the removal of senescent cells in a timely manner is crucial and pharmacological senolytic treatment using navitoclax has been shown to reduce hypertrophy and fibrosis in hearts from aged mice [204]. The SASP cells secrete a complex combination of growth factors such as GDF15, proteases, chemokines such as monocyte chemoattractant protein (MCP)-1, -2 and -4 and Mφ inflammatory protein (MIP)-1a and -3a, matrix metalloproteinases, and pro-inflammatory cytokines. They perpetuate a pro-inflammatory signaling loop and play a role in their own death, promoting the recruitment of immune cells, including Mφ which function collectively to clear the senescent cells (Figure 4). However, the immune response declines with age (“immunosenescence”), and, as a result, the clearance of senescent cells is impaired [205].

### 4.3. Protective Signals Favoring Proliferation of Resident Mφ

In the TAC model, resident Mφ initially proliferate and support angiogenesis in a KLF4-dependent manner [165]. This process has been proposed to be driven by renal CSF2 (colony stimulating factor 2) [164,165] (Figure 4).

In contrast, in response to β adrenergic-induced cardiac remodeling, our group recently reported that an early activation of the CX3CL1/CX3CR1 axis supported cardiac resident Mφ proliferation and delayed transition to HF [5] (Figure 4). This transient beneficial impact relied on the emergence of CX3CL1- and TNFα-cosecreting resident Mφ and their crosstalk with CX3CR1-expressing cardiomyocytes, leading to compensatory concentric hypertrophy [5]. Of note, CX3CL1 has also been previously described as a proangiogenic factor [134] (Figure 4).

### 4.4. Exosomes, Mir and Cardiac Mφ

Paracrine intercellular communications between cardiac cell types also occur via exosomes (secreted extravesicular vesicles) and the exchange of miRNAs (small noncoding RNAs that inhibit gene expression of complementary target genes at the posttranscriptional level). For example, Mφ exosome-derived miR-155 suppresses fibroblast proliferation, decreases collagen production promoting function alteration and cardiac rupture after MI [206]. It also favors pro-inflammatory Mφ polarization and cardiac monocyte infiltration inducing hypertrophy and failure in hypertensive models [207]. Mφ exosome-derived miR-21 drives pressure overload-induced cardiac fibrosis and dysfunction [208].

In contrast, recent literature highlights the concept that M2-exosomes-derived miR-24-3p targets the TNFα superfamily member Tnfsf10 (TRAIL) to reduce myocardial injury after sepsis, improving cardiac function [209] (Figure 4).

Promising results from preclinical studies point out treatments with miRNAs or antagomir deliveries as new potential therapeutic approaches to limit HF. For example, the use of a specific miR-21 antagomir allowed researchers to achieve indefinite cardiac allograft survival abrogating chronic allograft vasculopathy. Treatment with MiR-21 antagomir also led to a reprogramming of Mφ metabolism, with a shift toward oxidative phosphorylation, resulting in an increase in M2-like Mφ [210]. Similarly, miR-21 antagomir was shown to limit inflammation and attenuate histological and echocardiographic effects of experimental autoimmune myocarditis [211].

### 4.5. Immune Response and Fibrosis in Aging and Myocardial Diseases

Fibrosis may reflect activation of reparative or maladaptive processes. Because the adult mammalian heart has negligible regenerative capacity, death of a large number of cardiomyocytes results in reparative fibrosis, a process that is critical for preservation of the structural integrity of the infarcted ventricle. Pathophysiologic stimuli, such as pressure overload, volume overload, metabolic dysfunction, and aging may cause interstitial and perivascular fibrosis in the absence of infarction [212]. The potential protective role of replacement fibrosis to maintain cardiac function during the first steps of aging has been documented in EMMPRIN^−/−^ aged mice, (a matrix metalloprotease (MMP) inducer), that develop an aberrant extracellular matrix remodeling characterized by a loss of collagen deposition associated with a dilated cardiopathy as early as 12 months of age [213].

However, excessive cardiac fibrosis becomes a key driver of HF, a common pathophysiologic companion of most myocardial diseases, associated with systolic and diastolic dysfunction, aging, arrhythmogenesis, and adverse outcome [212].

For example, with aging and its associated evolution towards a low-grade oxygen environment, cardiomyocytes release pro-inflammatory cytokines and chemokines, stimulating an immune response. This leads to the increase in cardiac monocyte-derived CCR_2_^+^ Ly6C^high^ Mφ [31,214,215], referred as “inflammaging” [31,162,216], promoting fibrosis (Figure 5).

Cardiac Mφ secrete stromal cell proteins and are directly involved in ECM remodeling by producing inflammatory cytokines, TGFβ, PDGF, osteopontin, MMPs, and their inhibitors. They actively participate in the process of transformation of quiescent fibroblasts to myofibroblasts [135,212,217,218]. Anti-inflammatory cytokines, e.g., IL4 mainly secreted by resident Mφ, are also associated with profibrogenic properties. In the TAC model, IL4 neutralization attenuates fibrotic changes [219]. IL4 may exert direct fibrogenic actions by stimulating collagen synthesis in cardiac fibroblasts through activation of STAT6 [220]. Data concerning IL10 are conflicting with reported anti- and pro-fibrogenic related effects. It has been suggested that the final impact of IL10 may depend on the balance between anti-inflammatory and pro-fibrotic actions. For example, during the resolution phase of injury, “resolving” Mφ can secrete IL10 that exerts protective roles against cardiac fibrosis [212].

In patients with cardiomyopathy, CCR_2_^−^ Mφ seem to locate near the coronary vasculature, similarly to what has been reported for mice, whereas CCR_2_^+^ Mφ occupy fibrotic areas [161]. Cardiac-resident Mφ were reported to limit cardiac fibrosis in a pressure overload model [172] (Figure 4). Interestingly, Deniset et al. describe the pericardial cavity as an important source of resident Gata6^+^ Mφ that migrate into the heart, limit fibrosis of healthy myocardium, and improve functional cardiac recovery after ischemic injury, preventing detrimental repair caused by excessive fibrosis [221].

## 5. Future Directions

The overall analysis of the literature related to cardiac remodeling and transition to HF clearly outline the Janus nature of the inflammatory response, being either cytoprotective or detrimental, as well as the dynamic aspect of its impact. Therapeutic targeting of the NLRP_3_ inflammasome or of downstream IL1β signaling in patients with HF have been evaluated in clinical trials, making this pathway a promising target [222]. For example, the CANTOS study showed a significant reduction in HF-associated risk of hospitalization or HF-related mortality in patients treated with the IL1β inhibitor canakinumab [222,223]. In contrast, following the failure of global anti-TNFα strategies in HF patients, the development of novel classes of drugs selectively targeting TNFRs, e.g., selective blocking of sTNF/TNFR_1_ signaling which will preserve functional mTNF/TNFR_2_ signaling, or combination therapies using sTNF/TNFR_1_ antagonists together with TNFR_2_ agonists, might represent a novel superior therapeutic concept to treat a multitude of inflammatory and degenerative diseases including HF [224,225]. Supplementation in glutathione (with NAC) and/or inhibition of TACE activity might constitute additional valuable strategies to limit deleterious sTNF/TNFR_1_ signaling and promote TNFR_2_ pathways [72,73,101,108,226].

Cardiac Mφ are an emerging focus for therapeutic strategies aiming at strengthening adaptive responses, minimizing cardiomyocyte death, ameliorating pathological cardiac remodeling, and for treating HF [151]. Current knowledge clearly shows that the different cardiac Mφ populations are plastic, display various responses to injury, and differentially regulate inflammation and repair processes. Therapeutically, accumulating evidence indicates that strategies that will preserve or enhance the functions of CCR_2_^−^ Mφ and/or limit infiltration of CCR_2_^+^ monocytes, may provide additive benefit to established medications for HF. However, from studies examining cardiac remodeling after infarction, it clearly appears that keeping a time-dependent balance in the work of different subtypes of immune cells is crucial for successful heart healing and remodeling. In fact, the maintenance of early inflammatory activity is as important as the subsequent promotion of resolution and repair mechanisms after infarction [227]. In keeping, a timely planned targeting of specific subsets of Mφ will probably be necessary to achieve beneficial results in all types of cardiac pathologies. In this context, recent studies suggest that achieving the optimal recruited monocyte/resident Mφ loading after cardiac injury represents a therapeutic opportunity that might be achieved by targeting the cardiac lymphatic system to spatiotemporally constrain the innate immune response [163,195].

Recent transcriptomic and single-cell RNA sequencing studies allowed an evaluation of the progressive reprogramming of Mφ during cardiac remodeling. This led to the uncovering of potential specific properties of Mφ isolated from early adaptive vs. late failing hearts and to identify clusters of phagocytes with distinct gene expression profiles among which some are characterized by a mixed expression of pro-inflammatory and anti-inflammatory marker-genes, further emphasizing oversimplification of Mφ categorization into M_1_ and M_2_ cells [141,196,227,228,229]. Early adaptive resident Mφ amplified in response to β-AR stimulation were characterized by an induction of anti-inflammatory, pro-phagocytic and pro-angiogenic gene markers [141]. There is accumulating evidence that growth factor secretion plays a cardinal role in their protective impact in cardiac remodeling [230,231]. One of the modern concepts is that metabolic reprogramming of immune cells is a major factor of immune modulation, with oxidative phosphorylation and glycolysis promoting anti- and pro-inflammatory profiles, respectively [232,233,234]. In response to β-AR stimulation, early adaptive resident Mφ were characterized by an enrichment in genes related to oxidative mitochondrial phosphorylation, glucose and fatty acid oxidation, lipophagy, and Arginine signaling [141]. In addition, combined transcriptomic and lipidomic results showed a typical lipid remodeling with induction of genes coding for enzymes potentially leading to AA production and eicosanoid signaling [141]. In contrast, HF Mφ presented with an enrichment in glycolysis genes [141]. Among the many avenues that are suggested, such overall analyses may constitute the basis for more in-depth studies to further identify important Mφ-related pathways interfering in cardiac remodeling and/or characterize biomarkers associated with early vs. late disease progression. Current approaches using Mφ as therapies have essentially been developed in preclinical models mainly for rheumatoid arthritis and cancer uses, but some seem promising [235]. Targeting Mφ polarization might lead to novel intervention strategies in HF.

## Data Availability

No new data were created or analyzed in this study. Data sharing is not applicable to this article.

## Abbreviations

LV: left ventricular; HF, heart failure; AF, atrial fibrillation; HFpEF, Heart failure with preserved function; HFrEF, heart failure with reduced function; TAC, transverse aortic constriction; sTNFα, soluble tumor necrosis factor α; mTNFα, membranous tumor necrosis fact α; TACE, tumor necrosis factor α converting enzyme; TNFR_1_, Tumor necrosis factor receptor 1; TNFR_2_, Tumor necrosis factor receptor 2; TRADD, TNFR_1_-associated death domain; FADD, Fas-associated protein with death domain; RIP1/3, Receptor interacting protein 1/3; ROS, Reactive oxygen species; MLKL, Mixed lineage kinase domain like pseudokinase; MAPK, Mitogen-activated protein kinase; JNK, c-Jun N-terminal kinases; TRAF2, TNFR-associated factor 2; JAK, Janus kinase; ETK, Epithelial and endothelial tyrosine kinase; PI3K, Phosphoinositide 3 kinase; Akt, proteine kinase B; GSK3β, glycogen synthase kinase 3 β; STAT3, Signal transducer and activator of transcription 3; CX3CL1, Fractalkine; GSH, Glutathione; ROS, Reactive oxygen species; FAN, Factor associated with neutral sphingomyelinase activation; N-Smase, Neutral sphingomyelinase; RASSF1, Ras Association Domain Family Member 1; cPLA2, cytosolic phospholipase A2; AA, Arachidonic acid; LOX, Lipoxygenase, PI3K, Phosphoinosisitde 3 kinase; Akt, Proteine kinase B; ERK, Extracellular signal-regulated kinase; MSK1, Mitogen- and stress-activated kinase 1; CamKII, Calmoduline kinase II; PKC, Protein kinase C; Pi-PLB, Phosphorylated phospholamban; SAFE; CCR_2_, C-C motif chemokine receptor 2; Ly6c, lymphocyte antigen 6 complex; Ly6c, lymphocyte antigen 6 complex; IL4, Interleukin 4; IL10, Interleukin 10; GDF15, growth differentiation factors 15; KLF4, Kruppel-like factor 4; MERTK, myeloid-epithelial-reproductive tyrosine kinase; MDGF, myeloid derived growth factor; IGF1, insulin-like growth factor-1; TGFβ, Transforming growth factor β; MMPs, metalloproteinases; PDGF, Platelet-derived growth factor; VEGF, Vascular endothelial growth factor; FGF2, fibroblast growth factor 2; AREG, Amphiregulin; EGFR, Epidermal Growth Factor receptor; Cnx43, Connexin 43.
